# Nutritional Status and Health-Related Quality of Life among Home-Dwelling Older Adults Aged 75 Years: The PORI75 Study

**DOI:** 10.3390/nu16111713

**Published:** 2024-05-30

**Authors:** Susanna Kunvik, Jonna-Carita Kanninen, Anu Holm, Merja H. Suominen, Hannu Kautiainen, Juha Puustinen

**Affiliations:** 1 Faculty of Health and Welfare, Satakunta University of Applied Sciences, 28100 Pori, Finland; 2 Faculty of Technology, Satakunta University of Applied Sciences, 28100 Pori, Finland; 3 Faculty of Medicine, University of Turku, 20014 Turku, Finland; 4 Department of General Practice and Primary Health Care, Helsinki University Central Hospital, University of Helsinki, 00290 Helsinki, Finland; 5 Unit of Neurology, Satasairaala Central Hospital, Satakunta Wellbeing County, 28500 Pori, Finland

**Keywords:** nutrition, nutritional status, health-related quality of life, older adults

## Abstract

Malnutrition in older people has been considered as a health concern associated with a range of implications for health and functional ability. However, evidence of nutrition and health-related quality of life (HRQoL) among older people is limited. The aim of this study was to study the associations between nutritional status and HRQoL among home-dwelling older adults aged 75 years. In this cross-sectional study, we studied 75-year-old home-dwelling residents who participated in PORI75 preventive health screenings in 2020 and completed the full Mini Nutritional Assessment (MNA). The participants’ HRQoL was measured using the 15D instrument. Altogether, 462 participants (60% women) were included. Of these, 11% had decreased nutritional status (MNA score < 24); 12.7% were women and 8.6% were men, with no difference between the sexes (*p* = 0.17). A relationship was found between HRQoL and the MNA: a decreased MNA score was associated with decreased HRQoL (*p* < 0.001, r = 0.45, 95% CI: 0.38 to 0.53). All 15 HRQoL dimensions (except hearing) were associated with the MNA score. Among the men, the association was stronger compared to the women, especially when the MNA score was <24, indicating decreased nutritional status. In conclusion, impaired nutritional status seems to be associated with impaired HRQoL among 75-year-old people living at home, especially among men.

## 1. Introduction

The population aged ≥65 years is growing more rapidly than all other age groups [1]. In Finland, the proportion of people aged ≥65 years is expected to increase to 28% of the population by 2050. This demographic change is progressing so fast that Finland is ranked among the five fastest ageing populations in the world [1]. Prevention, supportive services and care are needed to improve the health and well-being of older people living at home. Adequate nutrition is important for maintaining good health, well-being, physical performance and quality of life [2,3,4,5,6,7]. However, ageing increases the risk of malnutrition due to many various factors, including physiological changes, social and environmental influences, decreased physical functioning, cognitive decline and multimorbidities [2,5,8].

Nutritional screening and assessment have been considered as important components of the multidimensional evaluation of older people [9]. Early detection and effective treatment can reduce the development of nutritional problems and associated negative health effects [10]. The most common validated screening tool for older people is the Mini Nutritional Assessment (MNA), which includes a short form (MNA-SF) and a full form (MNA-FF) [2,11]. It has been designed to provide a single, quick assessment of nutritional status among older people in different settings [11,12].

According to an international meta-analysis and systematic review, the prevalence of malnutrition according to the MNA in older people living at home was 3% and the risk of malnutrition was 27% [9]. The prevalence increases when the need for care increases. In a Finnish study of 400 older (≥75 years) people living at home and receiving home health care, 8% were found to be malnourished, and 86% were at risk of malnutrition according to the MNA [13].

Malnutrition in older people has been noted as a challenging health concern that is associated with physical decline, with wide-ranging implications for quality of life in general [8]. Older people with malnutrition are more likely to experience poor quality of life [14,15]. Maintaining a good nutritional status is the key element for better health and quality of life [8,16,17]. However, evidence of the associations between nutrition and quality of life among older home-dwelling people is limited.

The aim of this study was to study the associations between nutritional status and health-related quality of life (HRQoL) among home-dwelling older residents aged 75 years who participated in preventive PORI75 health screening in 2020. A secondary aim was to study the associations between nutritional status and the different dimensions of HRQoL in the 15D instrument.

## 2. Materials and Methods

The Social Security Centre of Pori has developed a health screening procedure for 75-year-old people to improve prevention and to improve the secondary use of patient data. The procedure has been described previously [18]. In this procedure, voluntary health screenings are provided every year to residents turning 75 years old. The health screenings are conducted in the Wellbeing Service County of Satakunta (formerly the Social Security Centre of Pori). In this cross-sectional study, we explored a sample of participants in the 2020 health screenings. The study has been approved by the Ethics Committee of the Hospital District of Southwest Finland.

All residents who turned 75 in the year 2020 and lived at their homes with or without home care were sent an invitation to participate for free in the PORI75 health screening. The invitation contained a pre-booked time for a nurse’s appointment, a request to answer the questionnaire before the appointment, information about laboratory tests and an informed consent document for participation in the study. The recruitment process was carried out between June 2020 and December 2020. Those residents were given written and oral information about the study protocol before giving informed consent. Written informed consent was received from all the volunteer participants personally or with the assistance of their caregiver or closest proxy.

**Figure 1:**
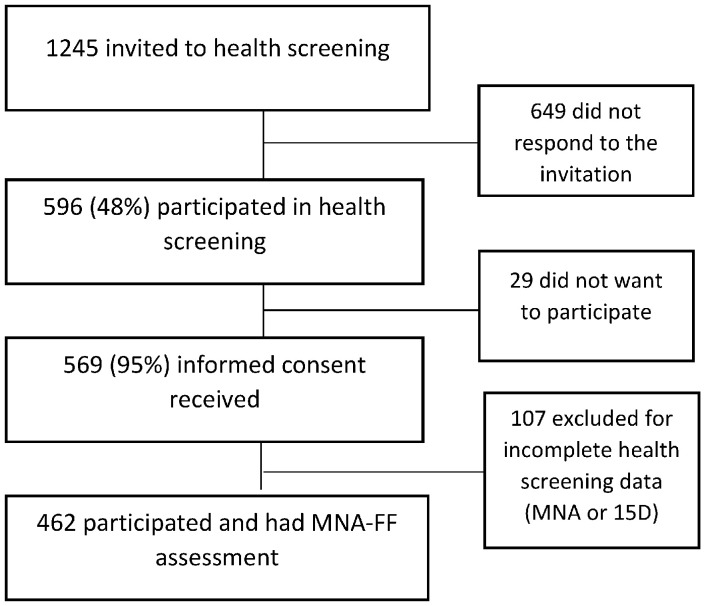
Study flowchart.

The recruitment procedure and sample number are described in Figure 1. Of the invited residents, 596 participated in the health screening. Of these, 569 (95%) gave written consent for their data to be included in the study, and 462 participants had complete health screening data, including a full Mini Nutritional Assessment (MNA-FF).

### 2.1. Measurements

The developed health screening procedure for 75-year-old people consisted of validated self-assessments, which the participants completed at home, and nurse-conducted screenings performed during the nurse’s appointment. The appointment lasted for two hours.

Self-assessments were posted by mail, and the participants returned their completed questionnaires at their appointment with the nurse. Activities of daily living (ADL) were used to measure daily activities [19]. The Geriatric Depression Scale (GDS-15) was used to assess depression [20], and the Simple Questionnaire to Rapidly Diagnose Sarcopenia (SARC-F) was used to measure the risk of sarcopenia [21]. Medication was measured from the self-reported questionnaire, and the total number of medications was computed using the anatomical therapeutic chemical (ATC) classification system, including medications A02–A16, B01–B06, C01–C10, D01–J07, L01–L04, M01–N07, P01–P03, R01–07 and S01–S03 [22]. Smoking was assessed from the self-reported questionnaire as “current smoker” or “non-smoker” (i.e., never having smoked or having stopped smoking). Special diets, such as a lactose-free or gluten-free diet, were assessed from the self-reported questionnaire as “yes, what special diet?” or “no”. The Alcohol Use Disorder Identification Test (AUDIT-C) was used to measure hazardous and harmful alcohol consumption [23]. HRQoL was assessed by the 15D questionnaire, which is a generic, self-administered questionnaire with 15 dimensions [24]. It consists of the following 15 dimensions: mobility, vision, hearing, breathing, sleeping, eating, speech, excretion (bowel habits and urination), usual activities, mental functioning, discomfort and symptoms, distress, depressive symptoms, sexual activity and vitality. Each dimension has five ordinal levels. The participant chooses from each dimension the level that best describes their health status. A set of utility or preference weights was used to generate the 15D score (i.e., a single index number) representing the overall quality of life on a scale of 0–1, where 1 represents the best quality of life [24].

Nurse-conducted screenings were performed during the nurse appointment. The full Mini Nutritional Assessment (MNA-FF) was used to screen for nutritional status [11]. A trained nurse interviewed the participants. The MNA includes 18 questions grouped into the four categories of anthropometry, general status, dietary habits and self-perceived health and nutrition states [12,25]. In this study, the MNA scores were divided into three categories based on the study results (dispersion of the data): <24 points, 24–26 points and >26 points. The first category was fewer than 24 points, and the participants with normal nutritional status were split into two to obtain an ordinal variable. The 5-item FRAIL scale (Fatigue, Resistance, Ambulation, Illnesses, and Loss of Weight) was used to screen persons at risk of developing disability, declining in health functioning, and mortality [26]. The score range is 0–5 (i.e., 1 point for each component; 0 = best to 5 = worst) and these scores represent frail (3–5), pre-frail (1–2) and robust (0) health statuses [26]. The Mini-Mental State Exam (MMSE) was used to assess cognitive functioning [27]. The Five Times Sit-to-Stand Test was used to measure functional lower-extremity strength, balance and transitional movements [28,29].

### 2.2. Statistical Methods

The study results are shown as numbers with percentages or as means with standard deviation (SD). The 95% confidence intervals are shown for the main outcomes. Statistical differences between the sexes were determined by t-tests, the chi-square test or Fisher’s exact test, as appropriate. Statistical significances for the hypothesis of linearity across ordered MNA scores were evaluated using the Cochran–Armitage test for trend, a linear-by-linear chi-square test and analysis of variance with an appropriate contrast. A possible nonlinear relationship between the MNA and HRQoL (15D score and dimensions) was assessed using a three-knot-restricted cubic spline regression model. The model was adjusted for sex and education. Crude and adjusted (partial) correlations were calculated using the Pearson method. The relationship between the MNA and the 15D was also analysed using a two-way analysis of variance. The models included the main effects (MNA score and sex) and the interaction effects between them. Correlation coefficients less than 0.2 were considered very weak, between 0.2 and 0.39 were weak, between 0.4 and 0.59 were moderate, between 0.6 and 0.79 were strong, and those above 0.79 were very strong [30]. *p*-values < 0.05 were considered statistically significant. The Stata 18.0 statistical package (StataCorp, College Station, Texas, TX, USA) was used for the statistical analyses.

## 3. Results

A total of 462 older adults (60% women) aged 75 who participated in the PORI75 health screenings in 2020 underwent the full MNA assessment. Differences were found between women and men in the baseline characteristics (Table 1). Fewer women were in a relationship compared to the men. More women than men had special diets, a higher number of medications, more depression symptoms (GDS-15) and a greater risk of sarcopenia (SARC-F). According to the AUDIT-C, the men had a greater risk of hazardous and harmful alcohol consumption than the women.

Both sexes had a good quality of life according to the 15D: the women had an average of 0.902 points, and the men had an average of 0.901 points, with no differences between the groups. However, there were differences between the sexes in the HRQoL dimensions (15D). The women had more points than the men in the dimensions of hearing, speech and sexual activity, thus showing a better quality of life in those dimensions. The men had a better quality of life in the 15D dimensions of sleeping, discomfort and symptoms, depression and distress.

A total of 11% (95% CI: 8.3–14.3) of the participants had a decreased nutritional status (MNA score < 24): 12.7% of the women (95% CI: 9.0–17.2) and 8.6% of the men (95% CI: 5.0–13.6), with no statistically significant difference between the groups (*p* = 0.17). The mean MNA score was 25.7 (SD 2.0) points. Most participants (48.3%) scored 24–26 points in the MNA.

Many background factors, such as having a special diet, the number of medications, an increased risk of depression (GDS-15), impaired ADL, decreased ability in the sit-to-stand test, risk of frailty and risk of sarcopenia, were associated with a risk of impaired (MNA score ≤ 26) nutritional status (Table 2).

**Figure 2:**
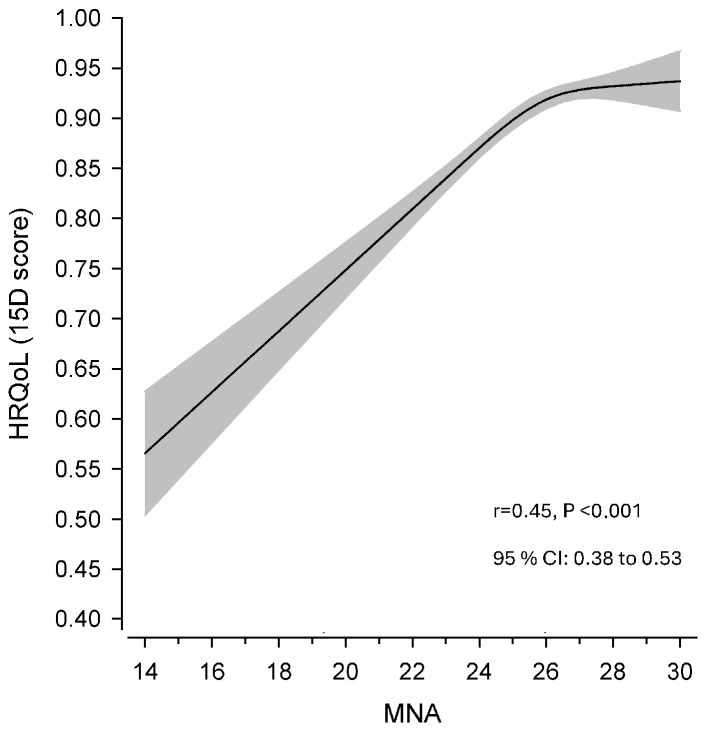
Relationship (Pearson correlation, r) between continuous Mini Nutritional Assessment (MNA) score and health-related quality of life (HRQoL, 15D). The curve was derived from a three-knot-restricted cubic spline regression. The model was adjusted for sex and education. The grey area shows 95% confidence intervals (CI).

A moderate relationship was found between HRQoL (15D) and the MNA: a decreased MNA score was associated with decreased HRQoL (Figure 2). The sex- and education-adjusted Pearson correlation (r) was 0.45 (95% Cl 0.34–0.55) and crude correlation was 0.45 (95% CI 0.38–0.53).

**Figure 3:**
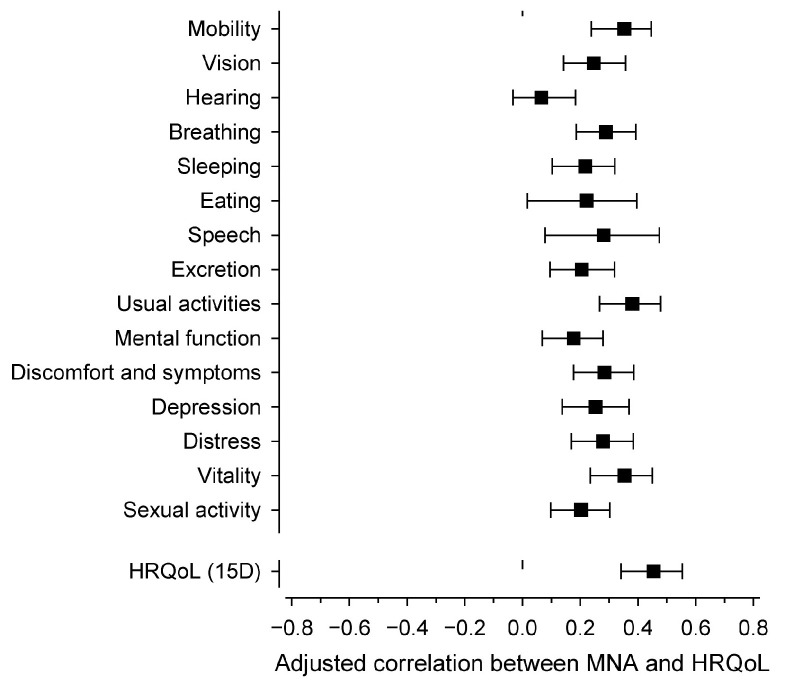
Sex- and education-adjusted (partial) correlations between the Mini Nutritional Assessment score and the health-related quality of life dimensions (15D). Whiskers show a 95% confidence interval. Correlation is statistically significant when whiskers do not intersect the dashed line.

The results from the partial correlation analysis between the MNA total score and the 15 dimensions are presented in Figure 3. All HRQoL dimensions (except hearing) were directly associated with the MNA score. The correlations for the 15D dimensions were quite similar, but slightly stronger correlations were found for mobility, usual activities, and vitality. However, the strongest correlation was with the total 15D score.

**Figure 4:**
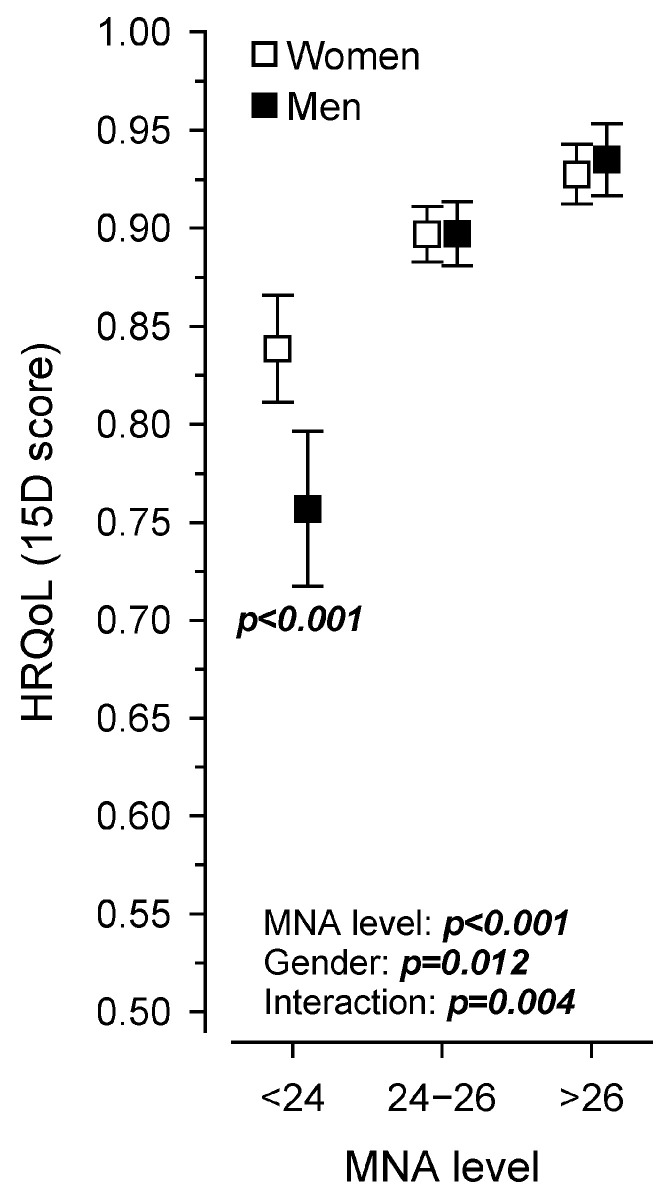
Health-related quality of life (15D) in men and women based on the Mini Nutritional Assessment score.

Figure 4 shows the relationship between the MNA score and HRQoL in men and women. Both the MNA score (*p* < 0.001) and the groups by sex (*p* = 0.012) were linearly associated with HRQoL, and the interaction was also significant (*p* = 0.004). Among the men, the association between nutritional status and HRQoL seemed to be stronger compared to the women, especially when the MNA score was under 24, indicating decreased nutritional status.

## 4. Discussion

In this study of home-dwelling 75-year-olds, we found that 11% had decreased nutritional status. Nutritional status was directly associated with HRQoL when the MNA score decreased to ≤26 points, showing that even a small decrease in the MNA score is associated with HRQoL. In addition, the MNA was directly correlated with all HRQoL dimensions (except hearing). Among the men, the relationship between nutritional status and HRQoL seemed to be stronger than among the women with a decreased nutritional status.

In this study, one in ten of 75-year-old residents had a decreased nutritional status, which seems to be lower than that usually seen in this population. In the systematic review and meta-analysis by Cereda et al. [9], an average of one-third of older people living at home had a decreased nutritional status. The PORI75 study sample may have included healthier individuals, as their HRQoL was better than the average older person living at home. This hypothesis is supported by the fact that the subjects’ HRQoL was better than average in comparison to the Finnish population aged 75+ years [31]. In addition, people attending preventive clinic visits may be more health conscious. The prevalence of malnutrition risk was similar to the 7.9% prevalence found among Swedish 75-year-old home-dwelling older people attending preventive clinic visits [32]. In our study, residents with a special diet, a large quantity of medication, depression, impaired daily performance and a risk of frailty or sarcopenia were more likely to have impaired nutritional status. These associations have been recognized in previous studies [2,33].

Although the number of residents with decreased nutritional status was relatively small, we were able to show a clear correlation between nutritional status and HRQoL. Similarly, a recent systematic review and meta-analysis showed a significant relationship between nutritional status and quality of life [16]. In our study, the correlation was shown when the MNA score was lower than 26 points, meaning that, even though there is not a nutritional risk according to MNA, a decreased score is related to HRQoL.

In our study, 14 out of 15 HRQoL dimensions (15D) were associated with nutritional status. Salminen et al. [34] also found that 12 out of 15 dimensions in the 15D instrument were associated with nutritional status among long-term care residents in Finland. This demonstrates the multidimensionality of the associations, covering a wide range of physical and psychological factors among older people from community through to long-term care settings. It remains unclear whether decreased nutritional status was the cause or the consequence or whether the association was mediated through a third possible factor, such as functional status. As HRQoL is one dimension of a wider concept of quality of life and is defined in relation to optimum levels of physical, mental and social functioning, the association covers a wide range of different factors which need to be considered [15]. For this reason, it is not enough to intervene only in older people’s nutrition. When nutritional status deteriorates, the root causes should be addressed from physical and psychological points of view.

In this study, the men showed a stronger relationship between nutritional status and HRQoL than the women when the MNA points were under 24 (i.e., indicating impaired nutritional status). This means that for men, even a slight deterioration in nutritional status appears to be linked to quality of life. To the best of our knowledge, this is the first study to show that nutritional status is associated with HRQoL among men even more than among women. These associations were not found in a previous Finnish study exploring the association between MNA and 15D measurements [34]. In addition, women have been more likely to report a lower quality of life than men [35]. In the present study, the women were more likely to report a lower physical health status, mental health status, cognitive function, social health status and quality of life than the men. The reasons for this difference should be considered. Men may have different causes of impaired nutritional status than women, leading to different impacts on quality of life. The reasons could be, for example, poorer cooking skills or psychosocial factors.

The strength of our study is the relatively large sample size of older residents living at home. The associations between nutritional status and HRQoL have usually been studied among hospital patients or frail people. Our study shows associations between nutrition and HRQoL among healthy 75-year-old residents living at home. However, this study has several limitations. Referring to the comparatively good MNA results, the population of this cross-sectional study may have been subject to selection bias and thus may not represent the average older person living at home. As is usually the case in voluntary health screenings, people already interested in their health and well-being participate. Those in poor health may have been excluded from the study. However, even though the population may have been selected, we were able to show the associations between nutritional status and HRQoL. It can be speculated how strong the correlations would have been with a population with more nutritional deterioration than in this study. Also, as the study took place in the early period of the COVID-19 pandemic, it may have affected the nutritional status and health-related quality of life of the participants. In addition, because of the cross-sectional study protocol, we could not show a causal relationship between nutrition and HRQoL. High-quality randomized controlled trials are needed to show the effect of nutritional status on HRQoL.

## 5. Conclusions

One in ten 75-year-old home-dwelling residents had decreased nutritional status, and several background factors were associated with it. Nutritional status was also associated with HRQoL, showing that even a small decrease in the MNA score was already associated with HRQoL. In addition, the MNA score was directly correlated with all HRQoL dimensions except hearing. Among men, the correlation between nutritional status and HRQoL seemed to be stronger than among women.

## Figures and Tables

**Table 1:** Background characteristics and health-related quality of life of women and men.

	Women *n* = 276	Men *n* = 186	*p*-Value
In a relationship, *n* (%)	143 (53)	149 (80)	<0.001
Education < 10 years, *n* (%)	196 (73)	137 (74)	0.71
Special diet, *n* (%)	45 (17)	16 (9)	0.014
Smoking, *n* (%)	13 (27)	18 (25)	0.88
AUDIT-C, points, mean (SD)	1.2 (1.2)	2.4 (2.1)	<0.001
Number of medications, mean (SD)	4.9 (3.0)	4.2 (2.8)	0.005
MMSE, points, mean (SD)	27.9 (2.2)	27.6 (2.4)	0.091
GDS-15, points, mean (SD)	5.7 (1.4)	5.4 (1.4)	0.010
ADL, points, mean (SD)	0.4 (1.5)	0.5 (1.7)	0.81
Sit-to-stand test, seconds, mean (SD)	15.3 (7.2)	15.7 (7.7)	0.58
FRAIL Scale, *n* (%)			0.97
Robust	206 (75)	141 (76)	
Pre-frail	58 (21)	39 (21)	
Frail	10 (4)	6 (3)	
SARC-F, points, mean (SD)	1.1 (1.6)	0.7(1.3)	0.017
15D, total score, points, mean (SD)	0.902 (0.079)	0.901 (0.099)	0.88
Mobility	0.911(0.168)	0.913 (0.158)	0.89
Vision	0.944 (0.136)	0.937 (0.141)	0.60
Hearing	0.943 (0.125)	0.915 (0.155)	0.038
Sleeping	0.791 (0.188)	0.852 (0.176)	<0.001
Eating	0.995 (0.046)	0.986 (0.082)	0.12
Speech	0.991 (0.050)	0.973 (0.105)	0.011
Excretion	0.834 (0.207)	0.853 (0.191)	0.32
Usual activities	0.919 (0.167)	0.889 (0.184)	0.076
Mental function	0.931 (0.145)	0.905 (0.172)	0.083
Discomfort and symptoms	0.761 (0.201)	0.809 (0.194)	0.011
Depression	0.921 (0.124)	0.945 (0.112)	0.034
Distress	0.908 (0.139)	0.939 (0.136)	0.017
Vitality	0.887 (0.135)	0.886 (0.138)	0.95
Sexual activity	0.911(0.207)	0.797(0.282)	<0.001

ADL: activities of daily living; AUDIT-C: Alcohol Use Disorders Identification Test-Consumption; FRAIL Scale: a short five-question assessment (fatigue, resistance, aerobic capacity, illnesses and loss of weight); GDS-15: Geriatric Depression Scale; MMSE: Mini-Mental State Exam; SARC-F: a simple questionnaire to rapidly diagnose sarcopenia; 15D: an instrument to assess the health-related quality of life; SD: standard deviation.

**Table 2:** Relationship between Mini Nutritional Assessment groups and background characteristics.

	MNA <24 *n* = 51	MNA 24–26 *n* = 223	MNA ≥26 *n* = 188	*p*-Value *
Women, *n* (%)	35 (69)	129 (58)	112 (60)	0.48
In a relationship, *n* (%)	9 (58)	141 (64)	122 (66)	0.30
Education <10 years, *n* (%)	42 (84)	162 (74)	129 (70)	0.080
Special diet, *n* (%)	11 (22)	32 (14)	20 (11)	0.048
Smoking, *n* (%)	4 (25)	11 (20)	16 (33)	0.25
AUDIT-C, points, mean (SD)	1.6 (2.0)	1.7 (1.6)	1.8 (1.8)	0.27
Number of medications, mean (SD)	6.6 (3.2)	5.0 (2.8)	3.7 (2.8)	<0.001
MMSE, points, mean (SD)	28.1 (2.6)	27.7 (2.3)	27.8 (2.1)	0.79
GDS-15, points, mean (SD)	6.2 (1.7)	5.6 (1.5)	5.4 (1.1)	<0.001
ADL, points, mean (SD)	1.6 (3.1)	0.5 (1.6)	0.1 (0.5)	<0.001
Sit-to-stand test, seconds, mean (SD)	18.2 (7.7)	15.5 (7.8)	14.8 (6.6)	0.012
FRAIL Scale, *n* (%)				<0.001
Robust	20 (39)	158 (71)	169 (90)	
Pre-frail	23 (45)	58 (26)	16 (9)	
Frail	8 (16)	6 (3)	2 (1)	
SARC-F, points, mean (SD)	2.0 (2.4)	1.0 (1.5)	0.6 (1.0)	<0.001

* Tests for trend across ordered MNA groups. ADL: activities of daily living; AUDIT-C: Alcohol Use Disorders Identification Test-Consumption; FRAIL Scale: a short five-question assessment (fatigue, resistance, aerobic capacity, illnesses and loss of weight); GDS-15: Geriatric Depression Scale; MMSE: Mini-Mental State Exam; SARC-F: a simple questionnaire to rapidly diagnose sarcopenia; SD: standard deviation.

## Data Availability

The data supporting this study’s findings are available from the Wellbeing Services County of Satakunta. Restrictions apply to the availability of these data, which were used under licence for this study. With the permission of the Wellbeing Services County of Satakunta, data are available from kirjaamo(at)sata.
